# The Tuberculosis Response and the Role of the BRICS in the Current Global Emergency

**DOI:** 10.1590/0037-8682-0216-2025

**Published:** 2025-08-08

**Authors:** Afranio Kritski, Ricardo Arcêncio, Ezio Tavora, Erica Chimara, Pedro Eduardo Almeida Silva, Jose Roberto Lapa e Silva, Martha Maria Oliveira, Monica Kramer de Andrade, Anete Trajman, Julio Croda, Maria Claudia Vater, Margareth Pretti Dalcolmo, Ethel Leonor Maciel

**Affiliations:** 1 Programa Academico de Tuberculose, Faculdade de Medicina, Universidade Federal do Rio de Janeiro, Rio de Janeiro, RJ, Brasil.; 2 Escola de Enfermagem de Ribeirão Preto, Universidade de São Paulo, São Paulo, SP, Brasil.; 3 Relações Internacionais e Projetos de Engajamento Comunitário em pesquisas da REDE-TB, Rio de Janeiro, RJ, Brasil.; 4 Núcleo de Tuberculose e Micobacterioses, Instituto Adolfo Lutz, São Paulo, SP, Brasil.; 5 Universidade Federal do Rio Grande, Rio Grande, RS, Brasil.; 6 Centro de Desenvolvimento Tecnologia em Saúde, Fundação Oswaldo Cruz, Rio de Janeiro, RJ, Brasil.; 7 Fundação Oswaldo Cruz Mato Grosso do Sul, Campo Grande, MS, Brasil.; 8 Faculdade de Medicina, Universidade Federal de Mato Grosso do Sul, Mato Grosso do Sul, MS, Brasil.; 9 Núcleo de Bioética e Ética Aplicada, Universidade Federal do Rio de Janeiro, Rio de Janeiro, RJ, Brasil.; 10 Escola Nacional de Saúde Pública, Fundação Oswaldo Cruz, Rio de Janeiro, RJ, Brasil.; 11 Universidade Federal do Espírito Santo, Vitória, ES, Brasil.; 12 Rede Brasileira de Pesquisa em Tuberculose, Rio de Janeiro, RJ, Brasil.

The BRICS country bloc - a cooperation among high-growth emerging economies, comprising Brazil, Russia, India, China, and South Africa - originated as “BRIC” in 2001, with the incorporation of South Africa in 2011. Although not a formal customs union like the European Union, the group holds annual ministerial and head-of-state summits and pursues a broad agenda, including economic and financial policies, food security, science, technology and innovation, and, increasingly, health, offering a South-South alternative to the Bretton Woods institutions. Indeed, in 2014, BRICS and partner nations launched the Shanghai-headquartered New Development Bank (NDB) and the Contingent Reserve Arrangement to fund infrastructure and sustainable development. 

Recently, in 2024, the bloc expanded to include seven new full members-Saudi Arabia, the United Arab Emirates, Egypt, Ethiopia, Iran, and Indonesia-forming the “BRICS+,” now representing roughly 40% of the world’s population and GDP. By rotating presidencies since 2008, BRICS has periodically redefined its global role. Brazil assumed the leadership of BRICS in 2025 and will has hosted the BRICS Heads of State meeting in Rio de Janeiro on July^1^.

To streamline collaboration, several thematic networks were established: four in education and three in science, technology, and innovation. The BRICS Tuberculosis Research Network, established in 2017, has led to significant research innovations in preventive measures, diagnostics, therapeutics, and public health. China, Russia, and India, for example, are currently leading domestic tuberculosis (TB) vaccine research and development (R&D), with four candidates in phase II/III trials as of 2024^2^. 

## A Preventable, Curable Disease as the Leading Global Killer

Despite being curable and preventable, TB remains the leading cause of death from a single infectious agent. In 2023 alone, 1.25 million people died-of whom 161,000 were HIV-infected-while 10.8 million fell ill and over 400,000 developed drug resistant (DR)-TB^3^. Nearly one-quarter of the world’s population is estimated to harbor latent TB infections. The BRICS nations account for 40% of global TB-related deaths and over 150,000 MDR-TB cases, representing 38% of the global burden. 

## Financing of TB services, research and innovation

The World Health Organization (WHO) has estimated that each United States dollar (US$) invested in TB elimination yields a US$ 43 return to society^4^.

In 2023, BRICS countries accounted for US$ 2.8 billion-63% of the total US$ 4.5 billion allocated domestically for tuberculosis in low- and middle-income countries (LMICs). Over the past decade, BRICS have invested more than US$ 20 billion in TB programs, research, and services, highlighting the importance of BRICS bloc´s strategy.

The WHO has recently (2023-2024) implemented a series of high-level actions to accelerate research and innovation, serving as the secretariat for the BRICS Tuberculosis Research Network^5^. 

Despite these achievements, a critical gap remains unaddressed: BRICS have yet to articulate a coordinated and accountable strategy to lead the development and adoption of transformative TB innovations. Given their scientific capacity and disease burden, BRICS nations carry not only the opportunity but also the obligation to drive the global agenda on new diagnostics, shorter treatment regimens, and effective vaccines. Without a concrete framework for innovation leadership and technology uptake, even the most promising tools risk failing to reach those who require them most.

The UN High-Level Meetings on TB in 2018 and 2023 secured commitments from member states to ramp up investments; however, these pledges have not translated into sufficient resources. 

## A Call for an Urgent BRICS Response

On May 14-15, 2025, Brazil convened the 18th Meeting of the BRICS Tuberculosis Research Network at a critical juncture for global TB elimination. Delegates called for more robust financing from the NDB to accelerate TB elimination through innovation: new vaccines, medicines, and diagnostics for both drug-susceptible (DS) and DR-TB, and strengthened prevention, diagnosis, and treatment strategies within national health systems. 

Dr. Tereza Kasaeva, Director of the WHO Global Tuberculosis Programme (GTB)^6^, cautioned that the recent withdrawal of funding from the United States Agency for International Development and the Centers for Disease Control and Prevention-which together account for 65% of the GTB’s annual US$15 million budget-poses a serious threat to the program’s continued operation. This would force the WHO to relocate the GTB from Geneva to the United Nations’s regional office. Without replacement funding in 2025, the world risks losing the GTB’s global mandate to set evidence-based guidelines, coordinate with multi-sectoral stakeholders, provide technical assistance, and monitor the epidemic (through annual reports from 200 countries and monthly surveillance of 100). The GTB also drives innovation, spearheading Artificial Intelligence-powered diagnostics, rapid tests, new vaccines, and shorter, more effective regimens for DS/DR-TB. All of these critical workstreams now face imminent collapse.

Proposals to relocate the GTB to a UN regional office would marginalize TB, crippling global coordination, responsiveness, and resource mobilization, with devastating consequences. 

It is imperative that Brazil, under its 2025 BRICS presidency, spearheads an emergency funding appeal: US$ 1.5 million annually from each of the original five BRICS members, and US$ 500,000 from each BRICS+ member to safeguard the GTB/WHO mandate. This modest investment, which is less than the cost of a large clinical trial, could save thousands of lives annually worldwide.

## Conclusions

The world expects BRICS to demonstrate collective leadership during global health emergencies. By mobilizing a coordinated response to tuberculosis, BRICS can reinforce its role as a key driver of equitable South-South cooperation and demonstrate that, with political will and strategic investment, TB elimination is an achievable goal.

The combined efforts of the BRICS countries could prevent 1.5 million TB deaths by 2030, provided investments are made in tandem with the sharing of technology. We must remember what happened to TB before it was declared a global emergency in 1993 when only two WHO teams were responsible for TB management. The COVID-19 pandemic has demonstrated the fragility of TB programs; temporary service disruptions have led to millions of missed or delayed diagnoses, highlighting how quickly the disease can re-emerge when programs falter. The collapse of surveillance systems has severely compromised the capacity of health authorities to detect, monitor, and respond effectively to disease outbreaks.

The withdrawal of global support for TB Control, Research and Innovation could trigger a preventable public health crisis and reverse decades of difficult progress achieved. Sustained investment in TB programs is not only a medical necessity but also a matter of global health security and social justice.

We urge governments, academic institutions, professional societies, and civil society movements to join this urgent campaign against the world’s oldest and deadliest infectious diseases. 
